# Genetic Determination and Linkage Mapping of *Plasmodium falciparum* Malaria Related Traits in Senegal

**DOI:** 10.1371/journal.pone.0002000

**Published:** 2008-04-23

**Authors:** Anavaj Sakuntabhai, Rokhaya Ndiaye, Isabelle Casadémont, Chayanon Peerapittayamonkol, Christophe Rogier, Patricia Tortevoye, Adama Tall, Richard Paul, Chairat Turbpaiboon, Waraphon Phimpraphi, Jean-Francois Trape, André Spiegel, Simon Heath, Odile Mercereau-Puijalon, Alioune Dieye, Cécile Julier

**Affiliations:** 1 Institut Pasteur, Unité de Génétique des Maladies Infectieuses et Autoimmunes, Paris, France; 2 INSERM U730, Paris, France; 3 Institut Pasteur, Laboratoire de Génétique de la réponse aux infections chez l'homme, Paris, France; 4 Institut Pasteur de Dakar, Unité d'Immunogénétique, Dakar, Sénégal; 5 Institut Pasteur de Dakar, Unité d'Epidémiologie, Dakar, Sénégal; 6 Institut de Médecine Tropicale du Service de Santé des Armées, Unité de Recherche en Biologie et épidémiologie parasitaires, IFR48, Le Pharo, Marseille, France; 7 Institut Pasteur, Unité d'Epidémiologie et physiopathologie des virus oncogènes, Paris, France; 8 Institut de Recherche pour le Développement, Laboratoire de Paludologie, BP 1386, Dakar, Sénégal; 9 Ecole du Val de Grace, Département d'Epidémiologie et de Santé Publique, Paris, France; 10 Centre National de Genotypage, Evry, France; 11 Institut Pasteur, Unité d'Immunologie Moléculaire des Parasites, CNRS URA 2581, Paris, France; National Institute of Neurological Disorders and Stroke, United States of America

## Abstract

*Plasmodium falciparum* malaria episodes may vary considerably in their severity and clinical manifestations. There is good evidence that host genetic factors contribute to this variability. To date, most genetic studies aiming at the identification of these genes have used a case/control study design for severe malaria, exploring specific candidate genes. Here, we performed a family-based genetic study of *falciparum* malaria related phenotypes in two independent longitudinal survey cohorts, as a first step towards the identification of genes and mechanisms involved in the outcome of infection. We studied two Senegalese villages, Dielmo and Ndiop that differ in ethnicity, malaria transmission and endemicity. We performed genome-scan linkage analysis of several malaria-related phenotypes both during clinical attacks and asymptomatic infection. We show evidence for a strong genetic contribution to both the number of clinical *falciparum* malaria attacks and the asymptomatic parasite density. The asymptomatic parasite density showed linkage to chromosome 5q31 (LOD = 2.26, empirical *p* = 0.0014, Dielmo), confirming previous findings in other studies. Suggestive linkage values were also obtained at three additional chromosome regions: the number of clinical malaria attacks on chromosome 5p15 (LOD = 2.57, empirical *p* = 0.001, Dielmo) and 13q13 (LOD = 2.37, empirical *p* = 0.0014 Dielmo), and the maximum parasite density during asymptomatic infection on chromosome 12q21 (LOD = 3.1, empirical *p*<10^−4^, Ndiop). While regions of linkage show little overlap with genes known to be involved in severe malaria, the four regions appear to overlap with regions linked to asthma or atopy related traits, suggesting that common immune related pathways may be involved.

## Introduction

Most malaria deaths occur as a consequence of complications following infection with *Plasmodium falciparum*. Severe malaria episodes may vary considerably in the nature of clinical manifestations, and in the event of death, its precise cause. Indeed, death may result from severe anaemia, cerebral malaria, acidosis or multiple organ failure, or a combination of these [1]. In addition, *P. falciparum* infection may be an indirect cause of death, which results from e.g. concomitant septicaemia. The contribution of host genetic factors to the risk of severe outcome following infection has long been recognized, with Haldane reporting for the first time a major role of one genetic variant in the β-globin gene, the sickle cell mutation (HbS), in the protection against severe malaria [2]. Since then, β -globin, and several other genes and genetic variants have been shown to be involved in the protection or susceptibility to severe malaria, including α-globin, HLA and several cytokine loci [3]]. Most of the protective variants are thought to have emerged in populations living in regions endemic for malaria as a result of the high selective pressure due to the parasite [2], [4]–[6]. In most cases, including HbS, the mechanisms underlying this protection remain unclear, as well as the role of particular genetic variants in the clinical manifestations of disease.

Most of the genes that have been explored and reported to date encode red blood cell proteins, proteins involved in the immune response to infection, or in other pathophysiological mechanisms directly relevant to malaria infection. These genes have been identified through case/control association studies, comparing severe malaria to uncomplicated malaria cases. Such a study design is limited in four aspects: 1) it has been restricted to the examination of specific candidate genes, based on their presumed functional relevance to malaria; 2) it provides little understanding of the causal role of specific genes and variants; 3) there is considerable variability among studies, depending on the disease selection criteria, population background and environmental context, which may result in poor reproducibility; 4) the severe *vs*. mild dichotomy is complex, because the mild malaria group may include patients who have not developed severe complications and yet are fully susceptible to severe malaria.

Evidence for a contribution of host genetic factors to phenotypes related to mild clinical malaria (such as number of clinical episodes, parasite density, immune responses to *P. falciparum* antigens) has been reported in four types of studies: concordance studies in monozygotic versus dizygotic twins [7], studies of two African sympatric ethnic groups differing in susceptibility to malaria [8], [9], segregation studies in malaria endemic populations [10], [11] and linkage analysis [12]–[15]. Several additional studies have shown association of specific genetic polymorphisms with clinical malaria [16]–[19]. However, these previous studies had limitations in the nature of the phenotypes that were considered and in the extent of genetic study. There is no clear picture of the mechanisms of naturally acquired immunity to malaria, and the relationship of mild to severe malaria is still unclear. Few studies have examined the genetic contribution to asymptomatic malaria [12]–[14], [20]. Recently, there has been a first attempt at a genome-wide linkage study approach that revealed several novel chromosomal regions linked to clinical and parasitological malaria traits [15].

In order to gain insight into disease mechanisms and the biological processes underlying the response to infection, and to identify the genes and genetic variants controlling these pathways, we designed a family-based genetic study of phenotypes related to infection with *P. falciparum*. This study was carried out in two independent villages, Dielmo and Ndiop that differ significantly in malaria transmission intensity and ethnicity in Senegal [21], [22]. Transmission in Dielmo is perennial and intense, while it is strictly seasonal and with a 10-fold lower number of infectious bites/year in Ndiop, so that exposure to infection and acquisition of immunity differ in both villages. Furthermore, the population of each village belongs to different ethnic groups. This study design enabled us to replicate the extent of genetic influence on malaria phenotypes identified in one village with the other. We recorded data related to malaria clinical attacks and asymptomatic infections for *P. falciparum* on a longitudinal, uninterrupted basis for over a decade [21], [22]. First, we defined phenotypes relevant to malaria infection that showed inter-individual variation, and we estimated the genetic contribution to these phenotypes. We then selected those phenotypes with significant genetic contribution for performing family-based genome-wide linkage studies.

This study enabled us to confirm the importance of a previously identified chromosomal region and identify two novel regions linked to the occurrence of clinical attacks and one novel region linked to asymptomatic parasite density.

## Results

### Family structure and ethnic groups

The entire population of each village was invited to participate in the study; there were no exclusion criteria. Less than 20% declined to participate. The majority of DNA samples were obtained from 3-generation families, though some were available from 4 generations of the families. The family structures were established using a questionnaire to the villagers, confirmed and adjusted by identity by state allele sharing of microsatellite genome-scan data in each pair of relatives. In each village, the majority of individuals were related to each other, forming one large complex family. Family structure statistics for each village are shown in Table 1. Within the large complex family, there are many small family units, some of which, because of multiple marriages, include several half-sibling relationships (sibships). Therefore, the small family units contained either (a) full-sibships only (FS), (b) mixed full- and half-sibships (MS), (c) half-sibships only (HS) or (d) only-child family (OC). Only individuals from whom we had DNA samples were included in the description of the family structure. When necessary, virtual individuals, which include absent or deceased relatives, were created to link the small family units. DNA samples were obtained from 421 individuals in Dielmo and 457 individuals in Ndiop. Thus, for the large complex family in Dielmo, there were 18 FS, 12 MS, 10 HS units and 52 OC, all of which were linked because at least one of the unit members was a 1^st^ cousin of someone else in this large complex family. There were 2 OC that were linked to the large complex family through a 2^nd^ cousin relationship and 2 FS units and 8 OC that were linked because they were uncles or aunts. For the large complex family in Ndiop, there were 20 FS, 15 MS, 6 HS units and 24 OC that were linked because at least one of the members was a 1^st^ cousin of someone else in this large complex family. There were 1 OC that was linked to the family through a 2^nd^ cousin relationship and 20 OC that were linked because they were uncles or aunts. In the MS units, the number of FS units varies. There are more FS units in MS units in Ndiop than Dielmo, which thus gave a higher number of half sib-pair counts in Ndiop (Table 1).

**Table 1:** Family structure and other characteristics of the two villages

	Dielmo	Ndiop
**Individuals characteristics**		
Sex: M/F ratio	1.24	1.11
Age^a^ median (min-max) yrs^b^	16 (0–83)	15 (0–76)
**Ethnic groups**	79% Serere	76% Wolof
	11% Mandinka	19% Fulani
	10% miscellaneous	5% miscellaneous
**Entomological Inoculation (infected bite/person)** c		
Rainy season (June–October)	132.5	23.8
Dry season (November–June)	60.1	0
**Family structures**		
Number of nuclear families	190	208
Number of independent families	10	21
Mean coefficient of inbreeding	0.0008	0.002
**Family size: number of individuals (number of families)**		
	453 (1)	503 (1)
	35 (1)	17 (1)
	30 (1)	14 (1)
	22 (1)	13 (3)
	11 (1)	12 (2)
	14 (2)	11 (2)
	8 (1)	9 (1)
	7 (1)	7 (1)
		6 (2)
		5 (3)
		4 (3)
		3 (3)
**Relative pair counts**		
Full sib-pair	513	547
Half sib-pair	299	780
Cousin pair	1157	1533
Parent-Child pair	842	924
Grandparent-Nephew pair	884	860
Avuncular pair	773	986

a Age at the first year of study

b 0 = newborn

c Average from 1990–99 in Dielmo, 1993–99 in Ndiop

In Dielmo, the ethnic groups consisted of 79% Serere (Niominka: 59% and Sine/Baol: 20%), 11% Mandinka and 10% miscellaneous, whereas in Ndiop, there were 76% Wolof, 19% Fulani and 5% miscellaneous.

### Malaria related phenotypes

For each person, the following malaria-related phenotypes were considered: 1) The number of clinical episodes of *P. falciparum* (acronym PFA). This phenotype characterizes the individual tendency to become clinically ill following *P. falciparum* infection; 2) The maximum and mean *P. falciparum* trophozoite parasite densities (i.e. pathogenic parasite stage infecting red blood cells) during clinical episodes (acronym mx-PFD and me-PFD, respectively). Maximum density was chosen to assess whether there were individual human differences in tolerance of parasite burden prior to onset of clinical symptoms. Because the parasite density eliciting a clinical reaction may also vary according to the parasite genotype, we considered a second related phenotype, the mean density; 3) The prevalence of asymptomatic *P. falciparum* infection, which reflects the acquisition of clinical immunity and/or the tolerance of parasitic infection (acronym PtPF). The acquisition of non-sterilising clinical immunity occurs with repeated exposure to the parasite. However, the rate of acquisition may also be influenced by human genetic differences independently of exposure; 4) The mean and maximum *P. falciparum* parasite densities during asymptomatic infection (acronym me-tPFD and mx-tPFD). These phenotypes are equivalent to those of category 2, but emphasising the ability of the individual (and human-parasite interaction) to tolerate parasite density without clinical reactivity.

For each malaria phenotype, we first performed statistical analyses of the data using multivariate regression analysis to take into account non-genetic factors such as age, year of study and transmission intensity (see Materials and Methods). The unexplained residual variation for each phenotype per person was then used for further analyses. As expected, some of the phenotypes were significantly correlated (Table 2). The residual number of clinical *P. falciparum* attacks (PFA) showed a significant positive correlation with the maximum parasite density during clinical attacks (mx-PFD) in both villages. It showed a moderate negative correlation with the mean and maximum parasite density during asymptomatic *P. falciparum* infection (me-tPFD and mx-tPFD, respectively) in both villages, before correction for multiple testing.

**Table 2:** Correlation of phenotypes

	Acronym a	PFA	me-PFD	mx-PFD	PtPF	mx-tPFD	me-tPFD	
NDIOP	PFA		**0.34**	**0.54**	**−0.23**	*−0.18*	**−0.21**	DIELMO
			**<0.0001**	**<0.0001**	**0.0009**	*0.01*	**0.002**	
	me-PFD	0.05		**0.68**	−0.14	−0.04	−0.13	
		0.43		**<0.0001**	0.22	0.76	0.26	
	mx-PFD	**0.35**	**0.64**		−0.11	−0.04	−0.13	
		**<0.0001**	**<0.0001**		0.22	0.68	0.14	
	PtPF	−0.08	−0.11	−0.06		**0.64**	**0.66**	
		0.15	0.11	0.36		**<0.0001**	**<0.0001**	
	mx-tPFD	−0.07	−0.14	0.03	**0.56**		**0.69**	
		0.27	0.054	0.69	**<0.0001**		**<0.0001**	
	me-tPFD	*−0.12*	−0.13	−0.01	**0.63**	**0.73**		
		*0.049*	0.08	0.90	**<0.0001**	**<0.0001**		

Regression coefficient shown above and *p* value below.

Results of Dielmo are shown in the upper right triangle and the results of Ndiop are shown in the lower left triangle. In bold, significance after Bonferoni multiple testing correction (*p*<.05), in italic, not significance (*p*>.05) after Bonferroni multiple testing correction (15 hypotheses tested per vilage). ^a^ For phenotype acronym, see Table 3.

### Genome scan linkage analysis

We selected phenotypes that showed significant heritability for further linkage studies (Table 3), namely: number of *P. falciparum* clinical attacks (PFA), the mean of parasite density during clinical attacks (me-PFD), prevalence of asymptomatic infection (PtPF) in both villages, the mean and the maximum (me-tPFD and mx-tPFD, respectively) asymptomatic parasite density. The mean asymptomatic parasite density (me-tPFD) was also studied in both villages despite the fact that it did not show a significant heritability in Ndiop, in order to enable comparison with previous studies [12]–[14]. The maximum parasite density during clinical attack (mx-PFD) was only studied in Ndiop because there was no significant heritability in Dielmo.

**Table 3:** Estimation of Heritability

Phenotype	Acronym	Dielmo	Ndiop
		*h^2^ (p-*value*)*	*h^2^ (p-*value*)*
Clinical attacks			
Number of clinical *falciparum* attacks	PFA	0.29 (0.0002)	0.27 (0.00002)
Mean parasite density (mean, Log10)	me-PFD	0.22 (0.03)	0.21 (0.02)
Maximum parasite density (max. Log10)	mx-PFD	0.02 (0.37)	0.13 (0.04)
Asymptomatic infection			
*P. falciparum* trophozoite			
Prevalence of asymptomatic PF infection	PtPF	0.29 (0.017)	0.35 (0.0001)
Asymptomatic trophozoite density of PF (mean, Log_10_ +1)	me-tPFD	0.33 (0.02)	0.07 (0.28)
Asymptomatic trophozoite density of PF (max., Log_10_ +1)	mx-tPFD	0.21 (0.04)	0.23 (0.01)

*h^2^* = heritability

Genome scan was carried out using 400 markers with average interval of 10 cM (Panel MD-10, Applied Biosystems). In addition, we typed 66 markers localised near candidate genes selected from studies of severe malaria (marker list available on request). These candidate genes were selected according to (i) their previous proposed association with severe malaria (e.g. *TNF*, NM 000594; *ICAM1*, NM_000201; *CD36*, NM 001001547, NM 001001548 and NM 000072; *CR1*, NM 000651 and NM 000573), (ii) their high frequency of mutations known to be common in malaria endemic region and suspected to be protective (e.g. *HBB*, NM_000518; *HBA1*, NM 000558 and *HBA2*, NM 000517; *SLC4A1*, NM 000342 [AE1 or band 3], *G6PD*, NM 000402 and NM 001042351) (iii) their role as a receptor for malaria antigens (e.g. the glycophorins) and (iv) their immunological response to malaria infection (e.g. *TGFB1*, NM 000660; FCGR gene family, *IL10*, NM 000572; *NOS2A*, NM 000625). An average information content of 0.68 (SD±0.12) was achieved at any point of the genome.

**Figure 1:**
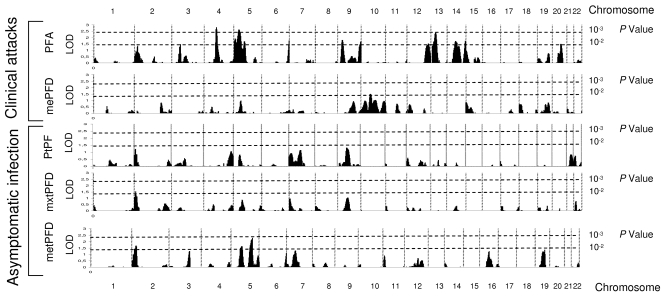
Genome scan linkage analysis results by the variance component method (SOLAR) in Dielmo, PFA, the number of clinical episodes of *P. falciparum*. mxPFD and mePFD, the maximum and mean *P. falciparum* parasite densities during clinical episodes. PtPF, the prevalence of asymptomatic *P. falciparum* infection. metPFD and mxtPFD, the mean and maximum *P. falciparum* parasite densities during asymptomatic infection. Vertical dotted lines represent chromosome boundaries, horizontal dotted lines indicate nominal *p* values corresponding to LOD score.

**Figure 2:**
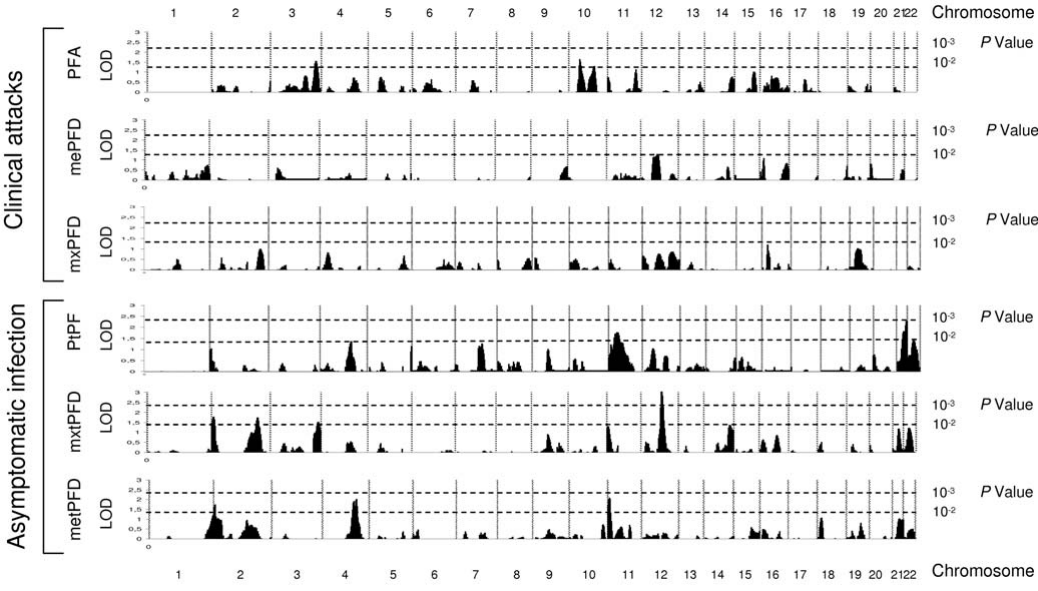
Genome scan linkage analysis results by the variance component method (SOLAR) in Ndiop, PFA, the number of clinical episodes of *P. falciparum*. mxPFD and mePFD, the maximum and mean *P. falciparum* parasite densities during clinical episodes. PtPF, the prevalence of asymptomatic *P. falciparum* infection. metPFD and mxtPFD, the mean and maximum *P. falciparum* parasite densities during asymptomatic infection. Vertical dotted lines represent chromosome boundaries, horizontal dotted lines indicate nominal *p* values corresponding to LOD score.

We performed duplicate linkage analyses using the variance component model (VCM) using SOLAR program [23] (see Materials and Methods), because of the robustness of this method in such a complex setting [24], and a regression linkage method using MERLIN program [25] (Regress, see Materials and Methods), which yielded overall comparable results. SOLAR can analyse large complex families such as observed here, while we modified the large complex family in each village into smaller sized ones in order to run MERLIN. The results of genome-wide analysis, using VCM, in the two villages are show in Figure 1 for Dielmo and Figure 2 for Ndiop. Several regions showed suggestive evidence of linkage, on the basis of LOD score values >2 with both VCM and Regress methods. Although several regions did show LOD score >2 by VCM, this result was not confirmed by Regress (e.g. Figure 1 Phenotype PFA chromosome 4). Indeed, only few regions reached such a LOD score value by both methods in each population studied.

**Figure 3:**
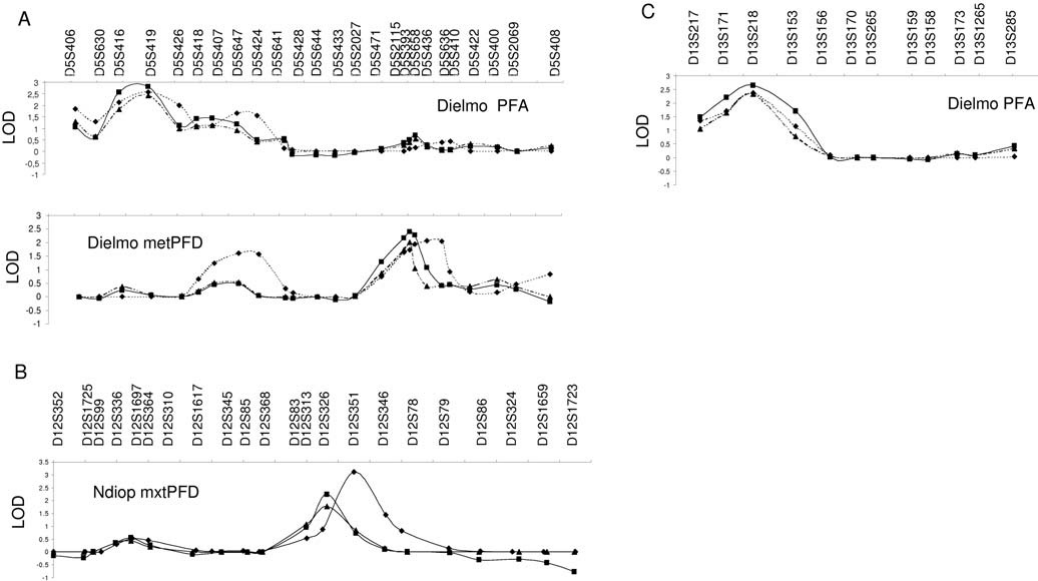
Detailed linkage analysis results with 2 methods: variance component (SOLAR and MERLIN) and regression (MERLIN); ⧫ for SOLAR variance component, ▪ for MERLIN regression, ▴ for MERLIN variance component. (A) Chromosome 5, (B) Chromosome 12, (C) Chromosome 13.

For the number of clinical *P. falciparum* attacks (PFA), linkage was detected in two chromosome regions in Dielmo: on chromosome 5p (near marker D5S419), with LOD scores of 2.57 (empirical *p* = 0.001) and 2.81 (nominal *p = *2×10^−4^) using VCM and Regress respectively (Figure 3A) and on chromosome 13q (near marker D13S218), with LOD scores of 2.37 (empirical *p* = 0.0014) and 2.65 (nominal *p* = 2×10^−4^) using VCM and Regress respectively (Figure 3C).

For asymptomatic *P. falciparum* infection, we observed linkage to the 5q31 region (near marker D5S436) with the mean transformed parasite density (me-tPFD) in Dielmo with LOD scores of 2.26 (empirical *p* = 0.0014, VCM) and 2.4 (nominal *p* = 4×10^−4^, Regress) (Figure 3A). In Ndiop, linkage to a region of chromosome 12 (near marker D12S351) was detected for the maximum of trophozoite density (mx-tPFD), with LOD scores of 3.1 (empirical *p*<10^−4^, VCM) and 2.27 (nominal *p* = 7×10^−4^, Regress) (Figure 3B). It is interesting to note that although the heritability of the mean *P. falciparum* asymptomatic trophozoite density (me-tPFD) did not reach significance in Ndiop, genome scan linkage analysis revealed a LOD score of 2.08 (empirical *p* = 0.005) by VCM and LOD = 1.06 (nominal *p* = 0.014) by regression analysis in the region of the β-globin cluster on chromosome 11p (Figure 2).

Interestingly, there was linkage to the same region of chromosome 2p with asymptomatic infection that was replicated in both villages with LOD scores >1. For mean asymptomatic parasite density (me-tPFD), we obtained LOD = 1.68, empirical *p* = 0.005 in Dielmo (near marker D2S168) and LOD = 1.75, empirical *p* = 0.0009 in Ndiop (near marker D2S319) by VCM in SOLAR and LOD = 2.16, empirical *p* = 0.0009 (near marker D2S319) when combining the 2 villages. LOD scores were similar with the maximum asymptomatic parasite density (mx-tPFD). This was not, however, reproduced when analysing with MERLIN that can not take into account large complex family structures.

## Discussion

The duration of the survey, which is the longest active case follow-up period to date for a genetic study of malaria [21], [22], allowed us to derive precise, robust phenotypes of clinical and asymptomatic *P. falciparum* malaria in both villages. With this study design, we were able to control for two major confounding factors of malaria infection rates, namely anti-malarial intake [26] and transmission intensity [27], [28]. Compared to previous linkage studies of clinical malaria [14], [15], our study is on a larger scale, with respect to the number of subjects and pairs of relatives. We show here that both clinical and asymptomatic malaria phenotypes were under genetic control in these populations. This genetic control includes the number of *P. falciparum* clinical attacks, *P. falciparum* density during clinical attacks and during asymptomatic infection. Based on genome-wide linkage analysis, suggestive evidence of linkage of these traits was detected in four chromosomal regions, including three novel ones.

In both villages, we found evidence for a strong genetic contribution to the residual number of *P. falciparum* clinical attacks (PFA). The heritability observed confirms previous observations in cohorts of Kenyan children [10]. It is remarkable that very similar heritability coefficients were estimated using two distinct study designs, recruitment procedures and, importantly, very different definitions of clinical malaria: fever associated with any *P. falciparum* density in Kenya [10] and fever associated with high, age-dependent parasite density in our study [29]. Interestingly, a strong genetic effect was detected in both villages for the prevalence of *P. falciparum* asymptomatic infection (PtPF) and maximal density of *P. falciparum* during asymptomatic infection (mx-tPFD). This suggests a genetic contribution to the control of peripheral blood parasitaemia. The equivalent measures for clinical episodes, PFA and mx-PFD, were positively associated with each other in both villages. This is consistent with the interpretation that the subjects experiencing many *P. falciparum* clinical attacks have a poor capacity to control parasite density, which frequently reaches the high threshold density necessary to elicit a clinical attack [29]. On the other hand, there was a negative correlation between the ability to harbour high parasite loads without symptoms (mx-tPFD and me-tPFD) and the number of clinical malaria attacks (PFA). Our results point to a genetic influence on the control of parasite density governing the occurrence of clinical attacks. In other words, higher asymptomatic parasite loads seemingly protect against occurrence of clinical episodes. One possible mechanism is through maintenance of an efficient concomitant “clinical” or anti-disease immunity reducing the risk of developing clinical malaria despite the presence of a relatively elevated parasite load. This is the first indication for a genetic basis to “premunition”, a combination of anti-parasite and clinical immunity acquired by individuals living in endemic areas [30].

We replicated the finding of linkage of the chromosome 5q31 region to *P. falciparum* asymptomatic parasite density that has been previously reported in three independent studies [12]–[14]. In Cameroon, Garcia and collaborators have detected suggestive linkage between 5q31 and malaria blood infection levels in 9 nuclear families [13], while in Burkina Faso linkage and association have been detected in much larger samples of 34 and 84 nuclear families [12], [14]. These results have been replicated in a malaria mouse model, *Plasmodium chabaudi*, where one quantitative trait locus for resistance to malaria has been mapped in homologous regions of human 5q31-q33 [31]. Compared to these previous studies, our study is more powerful in two ways. First the size of our population is higher (190 nuclear families in Dielmo and 208 in Ndiop). Second, the phenotype that we used for asymptomatic parasite density was derived from a much larger number of observations. We have used an average of 34 and 21 measurements per person in Dielmo and Ndiop, respectively while there were 6 and 20 measurements in Cameroon and Burkina Faso respectively [12]–[14]. In addition to malaria, 5q31 locus has been linked to schistosomiasis [32] and leishmaniasis [33], to several immune related disorders, including asthma/atopy [34], some autoimmune diseases such as inflammatory bowel disease [35], Crohn disease [36], Celiac disease [37] and psoriasis [38]. In addition, significant linkage to this region was shown in melanoma risk [39], autism [40] and schizophrenia [41]. This region contains a cluster of cytokines, which may represent strong candidate genes for control of malaria infection, but the causative gene(s) and variant(s), possibly common to all these diseases, remain to be identified. Among these genes, *IL12B* (NM_002187) seems to play a critical role since it has been associated with some immune-related diseases. An insertion/deletion polymorphism in the promoter region of *IL12B* has been reported to be associated with psoriasis [42] and cerebral malaria [43] while two intronic SNP polymorphisms were associated to asthma [44], [45]. However, we found no evidence of association of this promoter region polymorphism with the clinical malaria related phenotypes that we studied (data not shown).

With the exception of the β-globin locus, where we found suggestive evidence for linkage to some of the selected traits, there was no overlap of the regions of linkage that we detected and the location of the genes that have been previously reported to be associated with severe/cerebral malaria. We also did not find any evidence for linkage and association of the *ICAM-kilifi* variant with PFA and other malaria-related traits in these villages [46]. This apparent discordance between genes responsible for severe malaria and those controlling the response to *P. falciparum* infection in our study may reflect in part the constraints imposed by either approach, limited by the study of only selected candidate genes in previous studies, and limited in the power to detect small effects in our study. Moreover, variants responsible for susceptibility or protection against severe malaria, and that have a strong individual effect, may have a limited impact at the population level because of low frequency [47], as was suggested for β-globin in a Kenyan population [10]. The sample size of the populations studied here and the family structures are such that they are unlikely to present the entire repertoire of variants for malaria related genes. In addition, this may also indicate that the mechanisms (and genes) involved in the protection against severe malaria are largely independent of those involved in the response to mild clinical malaria and/or the control of blood parasitaemia.

Overall, genome scan linkage analyses showed peaks with higher LOD scores in Dielmo than in Ndiop. One reason for this is that the higher transmission intensity in Dielmo would reduce individual variation in exposure to infected bites, a major non-genetic factor. For asymptomatic phenotypes, prevalence (PtPF) gave lower LOD scores than parasite density (mean or maximum), although it showed highly significant heritability in Ndiop. Prevalence is strongly dependent on exposure and thus environmental factors, whereas parasite density will be determined by host-parasite biological interactions. Further analysis by the variance component method that included the effect of house supported this hypothesis: there was a strong effect of house on asymptomatic prevalence but not parasite density (data not shown).

We detected suggestive evidence of linkage to malaria-related traits in three new regions: chromosome 5p15-p13 and 13q13-q22 with the number of *P. falciparum* clinical malaria attacks (PFA) in Dielmo, and chromosome 12q21-q23 with the maximum parasite density during asymptomatic carriage (mx-tPFD) in Ndiop. Linkage results that we obtained in the two villages were notably different. This may be related to the important differences both in the ethnic backgrounds and in the prevailing transmission conditions in these villages. There was also no overlap between the regions that we detected in our study and those detected in an independent genome-wide linkage study of clinical *P. falciparum* malaria in Ghana [15]. Again, this may be explained in part by the different study design, as the Ghana study was restricted to children less than 12 years old, excluded any individuals with any of the major haemoglobinopathies (HbS, haemoglobin C, and alpha+ thalassaemia deletion 3.7) and/or with glucose 6 phosphate dehydrogenase deficiency A- and involved individuals with a different genetic background. On the basis of the “modest” value for linkage and multiple phenotypes under consideration, some of the regions detected with suggestive linkage in either study may also correspond to false positive results. Further genome linkage or association studies in other families and cohorts will be needed to confirm these initial results.

The three novel regions of suggestive linkage that we identified harbour a number of genes that can be considered as candidates for a role in malaria response. These include several genes involved in innate immunity such as interleukin [interleukin 7 receptor (*IL7R,* NM_002185)], tumour necrosis factor synthesis [C1q and tumor necrosis factor related protein 3 (*C1QTNF3,* NM_030945 and NM_181435)] and a gene involved in the complement system (*C9,* NM_001737). Genes located within these regions might participate in development of “clinical” immunity. Among these genes, *IL7R* (5p13) is implicated in B and T cell differentiation and is also critical for the innate and adaptive inflammatory and immunological response [48]. IL7 signalling is important for T cell differentiation of CD4– CD8– thymocytes and has a role in survival of CD4+ CD8+ cells after positive selection [48]. Several genetic and functional studies have associated genetic variants of *IL7R* gene with susceptibility to multiple sclerosis [49]-[51]. Therefore this gene could be a strong candidate for malaria infection. On the other hand, it has been reported that CD8+ T cells induced by *Plasmodium yoelii* sporozoites develop into protective memory cells without undergoing changes in interleukin-7 receptor α expression [52]. However, to date there has been no association study between *IL7R* polymorphisms and malaria, so further studies are needed to assess the implication of *IL7R* gene in malaria infection.

It is intriguing to note that these three regions of suggestive linkage, as well as the 5q31 region, where we replicated previous suggestive evidence of linkage, have all been previously found to be linked to asthma/atopic disease or related phenotypes [53]–[58]. Moreover, a mouse model for human atopic disease (NC/Jic) was found to be susceptible to murine malaria [59] and a major quantitative trait locus (*derm1*) for atopic disease mapped close to the region controlling parasitaemia (*char1* or *pymr*) on mouse chromosome 9 [60]. These findings raise the hypothesis that the development of clinical malaria may be due to an allergic reaction to malaria parasites or by-products of parasite infection, or that allergy/atopy and the response to malaria infection may share common mechanisms [61]. The observation that the number of clinical malaria attacks decreases with age and cumulative exposure in endemic areas, whereas the rate of asymptomatic infection increases, may reflect the development of immunotolerance rather than solely the development of immunoprotection. “Clinical” immunity to malaria may indeed be immunotolerance and absence of allergic-type responses rather than the presence of neutralising antibodies to malaria “toxins” as previously suggested [62], [63]. Several lines of evidence support the concept that susceptibility to malaria and atopy may be related to the same immunological defect. In Ethiopia, atopic children had a higher prevalence of malaria attacks [64], while in Tanzania maternal malaria had a protective effect on wheezing in children age of 4 [65]. The role of IgE in clinical and severe malaria is still poorly documented and results are controversial. *P. falciparum*-specific IgE is elevated in malaria patients and has been proposed to play a pathogenic role in severe malaria [66]–[68], whereas, in asymptomatic individuals, it was associated with protection [69]. An important role of the Th1/Th2 balance in the development of clinical malaria has been suggested by numerous studies [66].

Our study has limited power, as reflected by the relatively modest LOD scores. Indeed, similar limitations of linkage approaches have been encountered in most studies of multifactorial diseases, even when using much larger numbers of families [70]. The 10 cM linkage scan in our study provided an average of 70% coverage at any point along the genome. As far as linkage information is concerned, a slight increase in power could be gained using a higher density scan. However, this is unlikely to result in very different conclusions from the present work, which confirmed one previous reported linkage region and identified three new ones. On the other hand, much increased power would be expected using association studies with a higher density scan, as has been shown before [71]. One of the advantages of the family-based nature of the study is avoiding spurious association that may occur as a consequence of the population admixture that prevails in these villages, and generally in African villages. We are currently pursuing with a high-density SNP association screening in regions of linkage detected in this study in order to identify the loci involved in the control of the *falciparum* malaria related phenotypes investigated.

An additional limitation and one shared by all studies and approaches to date, is the accurate characterisation of disease phenotypes when dealing with a human population. General health status of the population, malnutrition and co-infection can alter the clinical outcome of infection [72], [73]. Helminth infestation in particular is widespread and, in Dielmo, has been suggested to adversely affect the clinical outcome of malaria infections [74]. Elimination of helminths can apparently reduce the risk of severe disease in areas with stable and endemic malaria [74] but has no such effect in areas of low, unstable transmission [75], [76]. Inclusion of all such confounding factors in our analyses would require an unworkably complex study design and statistical analyses.

Our study provided several new and interesting findings, not only on the genetic basis to disease but also on how human genetics may influence the biology of the parasite within the host. We defined relevant malaria related traits and estimated their genetic contribution, and mapped genes involved in their control. In addition to replicating linkage to the 5q31, and to some extent to the β-globin locus, we identified linkage to three additional regions. While these regions are extensive and contain many putative candidate genes, it is remarkable that 5q31 and these three novel regions overlap with regions that have been previously identified to be involved in asthma/atopy, hence suggesting that common mechanisms may be involved between both pathogenic mechanisms. These results will provide highly valuable information in the context of integrated studies combining linkage and high density association studies for various malaria traits and conditions. Ultimately, these results will help understand the molecular mechanisms underlying pathogenesis, control of parasite density, and possibly immunotolerance to malaria antigens and the potential relationship with the asthma/atopic disease spectrum.

## Materials and Methods

### Subjects

The Dielmo and Ndiop longitudinal surveys have been described in detail elsewhere [21], [22]. The project protocol and objectives were carefully explained to the assembled village population and informed consent was individually obtained from all subjects either by signature or by thumbprint on a voluntary consent form written in both French and in Wolof, the local language. Such consent was obtained in the presence of the school director, an independent witness. For very young children, parents or designated tutors signed on their behalf. The protocol was approved by the Ethical Committee of the Institut Pasteur de Dakar and the Ministère de la Santé of Senegal. An agreement between Institut Pasteur de Dakar, Institut de recherche pour le développement (IRD) and the Ministère de la Santé et de la Prévention of Senegal defines all research activities in Dielmo and Ndiop villagers. Each year, the project was re-examined by the Conseil de Perfectionnement de l'Institut Pasteur de Dakar and the assembled village population; informed consent was individually renewed from all subjects. DNA samples were obtained from 421 individuals in Dielmo and 457 individuals in Ndiop.

### Study sites

Two villages of differing malaria epidemiology were studied. Malaria transmission is perennial in Dielmo, where a river maintains larval breeding sites for the mosquitoes. By contrast, malaria transmission is strictly seasonal in Ndiop and dependent upon the rainy season that occurs from July-September. Such differing transmission has marked consequences on the epidemiology of malaria in the villages. This is most evident in the higher *P. falciparum* prevalence rates of infection in Dielmo (80+%) compared to the seasonal rates in Ndiop that change from 20% in the dry season to 70% in the rainy season [22], [77]. Peak incidence rates of clinical disease due to *P. falciparum* occur in very young children (2–5 years old) in Dielmo and then decline rapidly with age compared to 5–10 years old in Ndiop with only a gradual decrease with age [78]. The acquisition of non-sterilising clinical immunity thus generates a much higher prevalence of asymptomatic infections in Dielmo. A field research station with a dispensary was built for the project in each village to identify (and treat) all episodes of morbidity. Similar entomological surveys and identical strict clinical surveillance programs were carried out in both villages. The level of malaria transmission was monitored during the whole study period. Night-time collections of mosquitoes landing on volunteer subjects living in Dielmo and Ndiop were carried out weekly or monthly. It was thus possible to estimate the entomological inoculation rate (EIR), i.e. the number of infective bites, for every time period. During the study, Dielmo had a ten-fold higher EIR than Ndiop (Mann-Whitney *p* <0.001).

### Malaria related parameters measurement

During the first intensive survey period, malaria parasitaemia was determined by thick blood film for each inhabitant regardless of symptoms, twice a week from June to September 1990 in Dielmo and once a week from July 1993 to January 1994, then once a month from January to July 1994 in Ndiop. All thick blood films were examined by reading 200 oil-immersion fields on each slide (about 0.5 µL of blood) [79]. The ratio of *P. falciparum* trophozoites to leukocytes was established. Measures of parasitaemia i) during pregnancy and ii) in the three days preceding or following any fever episode were excluded from the analysis of asymptomatic parasitaemia. Only individuals with at least six eligible estimations of parasitaemia were considered.

Following this initial period, only clinical malaria attacks were recorded. Case detection was both active and passive. A villager, who had volunteered to participate in the study, was visited daily at home by a physician, a technician, a nurse or medical field workers. Thick blood films were prepared and detailed medical examinations were made for villagers who had fever or any symptom that could be related to clinical malaria. A standardized questionnaire was completed for each episode of illness, recording clinical findings, treatment administered [80] and response to treatment. None of the inhabitants received antimalarial chemoprophylaxis. The justification for not providing chemoprophylaxis within the project was based on the semi-immune status of the population, the level of chloroquine resistance in the area, and the permanent presence of a medical team in each village with daily home visits to each inhabitant. Urine tests were regularly carried out in the villagers to detect the presence of antimalarial drugs. Less than 2 percent of the results of tests carried out during the study period were compatible with unprescribed self-treatment. We considered an episode of *P. falciparum* clinical malaria in any case of fever (axillary temperature > = 37.5°C) or fever-related symptoms (headache, vomiting, subjective sensation of fever) associated with i) a *P. falciparum* parasite/leukocyte ratio higher than an age-dependent pyrogenic threshold previously identified in the patients from Dielmo [29] or ii) a *P. falciparum* parasite/leukocyte ratio higher than 0.3 parasite/leukocyte in Ndiop.

The survey periods were defined by trimester for each inhabitant. Only person-trimesters with more than 30 days under survey in the village were included in the analysis. Person-trimesters with pregnancy during the trimester were excluded.

### Transformation of data

Data for each malaria-related phenotype were first analysed using multivariate regression analysis according to the distribution of the data. This method enabled us to take into account known confounding factors such as age, year of study and transmission intensity. The residual unexplained variation in each phenotype was then calculated from the difference between the observed and expected values and then used as the phenotype for further genetic analysis.

For clinical malaria attacks, we considered the number of clinical malaria attacks recorded during June 1990 to May 1998 in Dielmo and during July 1993 to May 1999 in Ndiop. The median (range) number of clinical *P. falciparum* malaria attack/person was 1 (0–69) in Dielmo and 2 (0–37) in Ndiop. The residual number of clinical *P. falciparum* attacks was transformed from the number of clinical malaria attacks during person-trimesters separately for each village by Poisson regression models including the effect of age, level of transmission and year of survey. To take into account the malaria exposure dependent acquired immunity in Dielmo, the models also controlled the effect of the proportion of time spent in the village for each individual from his/her birth to the beginning of person-trimester survey period. This confounding factor had no significant effect in Ndiop. Mean and maximum of decimal log transformed maximum parasite density during clinical attacks were analyzed as residuals after accounting for age using linear regression.

Asymptomatic parasitaemia was evaluated from the systematic thick blood films collected from each inhabitant during the first intensive survey period as described above. The median (range) number of measurements per individual was 33 (6–46) in Dielmo and 24 (6–34) in Ndiop. Asymptomatic parasitaemia was considered in two respects, namely prevalence and intensity. For the prevalence, the residual risk of having a positive blood smear for trophozoites was estimated. First, the probability of having a positive slide was separately estimated for trophozoites by logistic regression models, including variables for the effect of age, week of sampling and delay between the end of the preceding anti-malarial treatment and the date of sampling for each thick blood film. For each individual, the expected number of positive thick blood films was calculated by the sum of the probabilities estimated by the models divided by all observations of parasitaemia eligible for analysis from that individual. An Anscombe residual was then calculated for each individual as a function of the sum of the observed and the expected positive thick blood films. Densities were estimated as the number of trophozoites per 100 leukocytes. Addition of 1 to the density before decimal logarithm transformation allowed negative blood films to be taken into account. The expected parasite density of all thick blood films was estimated by multiple linear regression analysis controlling for the same confounding factors as for prevalence. A raw residual was calculated by subtracting the observed from the expected parasite density. The mean raw residual was calculated for each individual and was used as a phenotype for further genetic analyses. In order to test the maximum capacity of each individual to carry parasites without symptoms, we considered the maximum parasite density of all measurements during asymptomatic carriage. We performed decimal logarithm transformation and controlled for age and week of study.

The transformed phenotypes were tested for normal distribution by estimation of skewness and kurtosis and Shapiro-Wilks test for normality. All phenotype analyses were performed using STATA version 7.

### Microsatellite Genotyping

DNA was extracted from 10 mL venous blood or 0.5 mL capillary samples and randomly amplified using a primer extension pre-amplification method [81]. Genotyping of microsatellites was carried out using a ABI377 system (Applied Biosystems, Foster City, USA). Genotyping results were checked for excess homozygotes or excess heterozygotes assuming Hardy-Weinberg equilibrium. Markers, which showed excessive homo- or heterozygosity were re-examined. Mendelian inheritance within families was confirmed using the PedCheck program [82] and inconsistencies resolved by re-examination of the raw data and re-genotyping where necessary. After Mendelian inheritance was resolved, unlikely genotypes were detected by the program MERLIN. Suggested microsatellite genotyping errors were corrected by re-examination of the raw data and re-genotyping where necessary.

### Statistical genetic analysis

Pedigree structures were checked by identity by state allele sharing of each relative pair from genotyping results of microsatellite markers used during genome screening using IBS_check program (Heath, unpublished).

Evaluation of the genetic contribution to the phenotypes was performed by estimation of the heritability using the variance-component model [83]. A variance components analysis of family data decomposes the total variance of the phenotypes into 3 components that are due to genetic (polygenic) (*h^2^*), individual (*e^2^*) and quantitative trait locus (*q^2^*) effects. First, we tested for heritability of the phenotype by comparing likelihood between the reduced model, where total variation is due to environmental variation only, and the full model where total variation is composed of environmental and genetic effects estimated from the genetic relationship of each pair of individuals. When the null hypothesis was rejected, heritability (*h^2^*) was then estimated as the percentage of genetic variance of the total.

For phenotypes that showed significant heritability, we performed linkage analysis to estimate the genetic variance attributable to the region around a specific genetic marker [83]–[86]. We compared the reduced model where total variation is due to environment and polygenic with the full model that included the locus specific effect using identity by descent estimation from the corresponding genetic marker.

Estimation of heritability and linkage analyses were run by use of the SOLAR program (version 2.1.3). SOLAR can analyse large complex families such as observed here. The complex family structure is accounted for in the genetic analysis via the polygenic variance component that uses the relationship coefficient between pairs of individuals as a parameter [83]. As several phenotypes showed residual kurtosis of more than 0.8, t option, which creates an extra parameter in the model to describe the distribution of the phenotype, was applied in all analyses. A general additive model was used, making no assumption of dominance or recessive nature of the gene. Empirical *p* values were obtained by performing a simulation of 10,000 trials implemented in the SOLAR software [23], [24]. Identity by descent status for each locus was estimated for the linkage study as a multipoint fashion using Markov Chain Monte Carlo methods by use of LOKI (version 2.4.5). SOLAR allows us to include house (shared environment) variance component in the model by creating a house matrix including pairs of individuals across the populations, if they are in the same house, they will be assigned a value of 1 and if not, 0.

In addition to variance component analysis using SOLAR, we performed regression linkage and variance component analysis using the MERLIN program [25]. The regression linkage analysis used a regression-based procedure for linkage analysis that uses trait-squared sums and differences to predict identity by descent sharing between any non-inbred relative [87]. In order to run MERLIN, we modified the large complex family in each village into smaller sized ones. In the simplified family structure, each individual will have only 1 sibling relationship. When these individuals occur in another pedigree, they will be parents or grandparents of the family without phenotypes.
