# Correction: Genetic Determination and Linkage Mapping of *Plasmodium falciparum* Malaria Related Traits in Senegal

**DOI:** 10.1371/annotation/d0a416fa-b683-4721-88c1-3f6dc9a04d8d

**Published:** 2008-04-30

**Authors:** Anavaj Sakuntabhai, Rokhaya Ndiaye, Isabelle Casadémont, Chayanon Peerapittayamongkol, Christophe Rogier, Patricia Tortevoye, Adama Tall, Richard Paul, Chairat Turbpaiboon, Waraphon Phimpraphi, Jean-Francois Trape, André Spiegel, Simon Heath, Odile Mercereau-Puijalon, Alioune Dieye, Cécile Julier

There was an error in the fourth author's name. It should read "Chayanon Peerapittayamongkol."

